# Supplementary material for: Substrate-induced assembly and functional mechanism of the membrane protein insertase SecYEG-YidC
Source: EMBO J. 2026 Jun 26;45(15):5457–81. doi: 10.1038/s44318-026-00837-6 (PMC13434473; doi:10.1038/s44318-026-00837-6)
Supplement: Supplementary file 1 [file 44318_2026_837_MOESM10_ESM.pdf]

Expanded View Figures

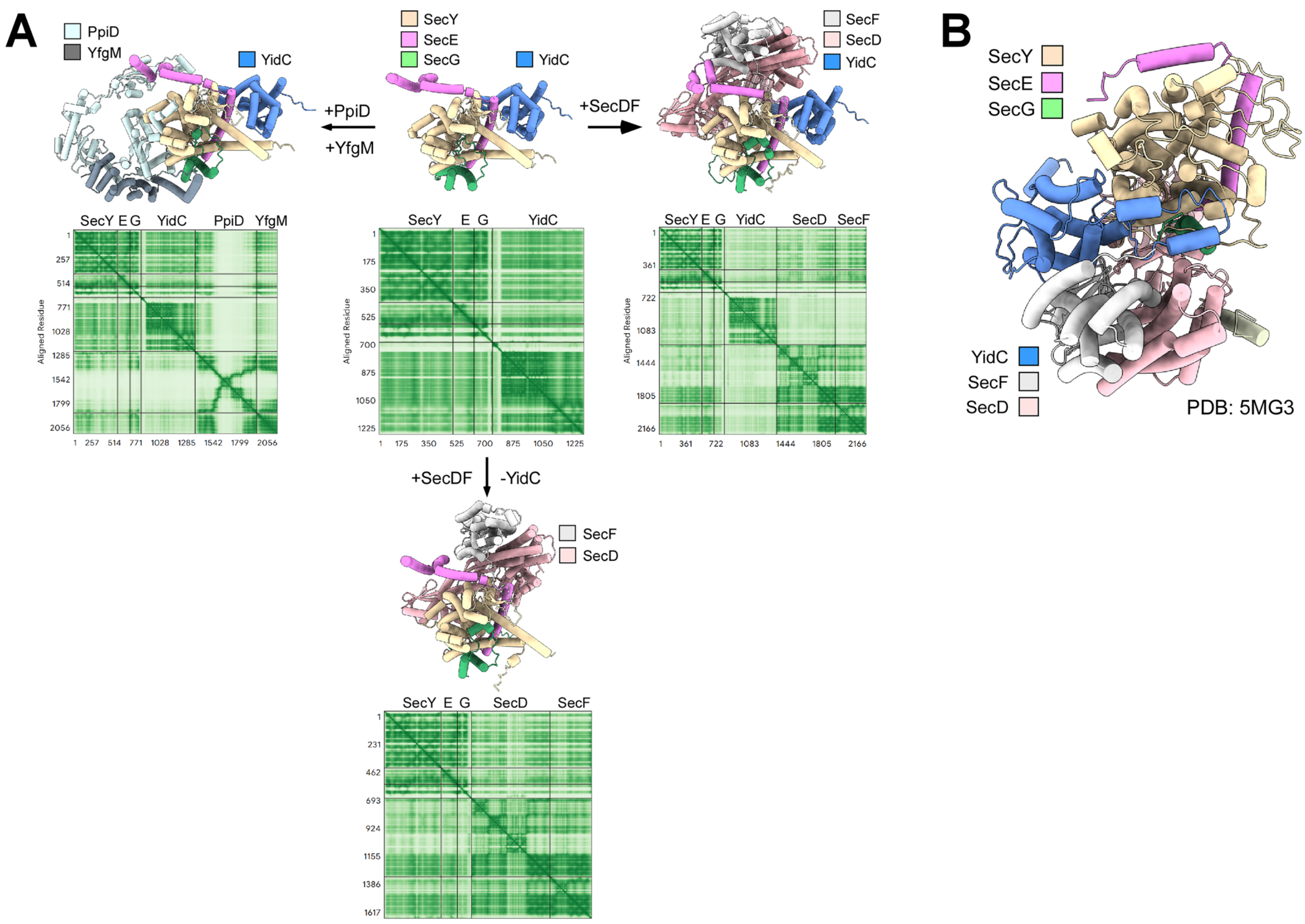

Figure EV1. Potential interaction network of *E. coli* SecYEG.

(A) AlphaFold3-based models and PAE plots of putative complexes formed by *E. coli* SecYEG with the accessory proteins. YidC is docked at the back of SecYEG with high confidence (low PAE levels), though co-assembly with SecDF negatively affects the model confidence for both YidC and SecDF. This may be explained by steric hindrance and partial overlap of the binding sites at SecYEG. Single-span protein YajC manifested random positions between the models, with high PAE scores, so it was excluded from analysis. The generated AlphaFold models are provided in the Dataset EV1. **(B)** Model of the SecYEG-YidC-SecDF assembly in detergent, based on the low-resolution cryo-EM reconstruction (PDB ID 5MG3).

 © The Author(s)

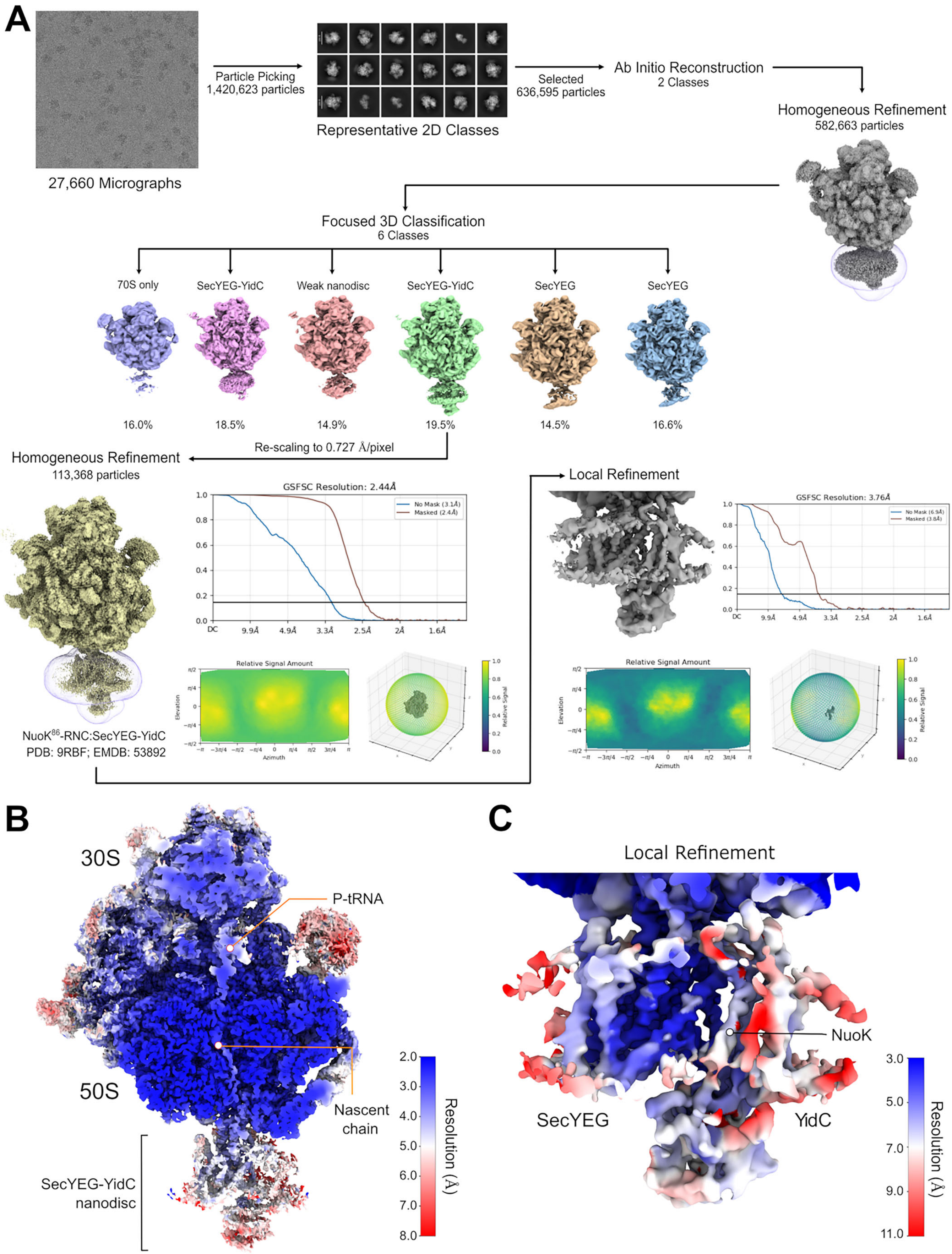
A
27,660 Micrographs
Particle Picking
1,420,623 particles
Representative 2D Classes
Selected
636,595 particles
Ab Initio Reconstruction
2 Classes
Homogeneous Refinement
582,663 particles
Focused 3D Classification
6 Classes
70S only
SecYEG-YidC
Weak nanodisc
SecYEG-YidC
SecYEG
SecYEG
16.0%
18.5%
14.9%
19.5%
14.5%
16.6%
Re-scaling to 0.727 Å/pixel
Homogeneous Refinement
113,368 particles
GSFSC Resolution: 2.44Å
No Mask (3.1Å)
Masked (2.4Å)
Relative Signal Amount
Elevation
Azimuth
Relative Signal
NuoK86-RNC:SecYEG-YidC
PDB: 9RBF; EMDB: 53892
Local Refinement
GSFSC Resolution: 3.76Å
No Mask (6.9Å)
Masked (3.8Å)
B
30S
P-tRNA
50S
Nascent
chain
SecYEG-YidC
nanodisc
Resolution (Å)
C
Local Refinement
NuoK
SecYEG
YidC
Resolution (Å)

Figure EV2. Cryo-EM data analysis, classification and resolution of the cross-linked NuoK^{86}-RNC:SecYEG-YidC complex.

(**A**) Classification and refinement scheme employed for single-particle analysis. The structurally distinct classes, distribution of views and Fourier shell correlation plots are shown. (**B**) Globally refined cryo-EM map for the main SecYEG-YidC class, with the color-code according to the local resolution. Gold-standard Fourier shell correlation (GS-FSC) curve as well as angular distribution plot are shown. (**C**) Locally refined cryo-EM map of the nanodisc-embedded insertase with the color-code according to the local resolution.

© The Author(s)

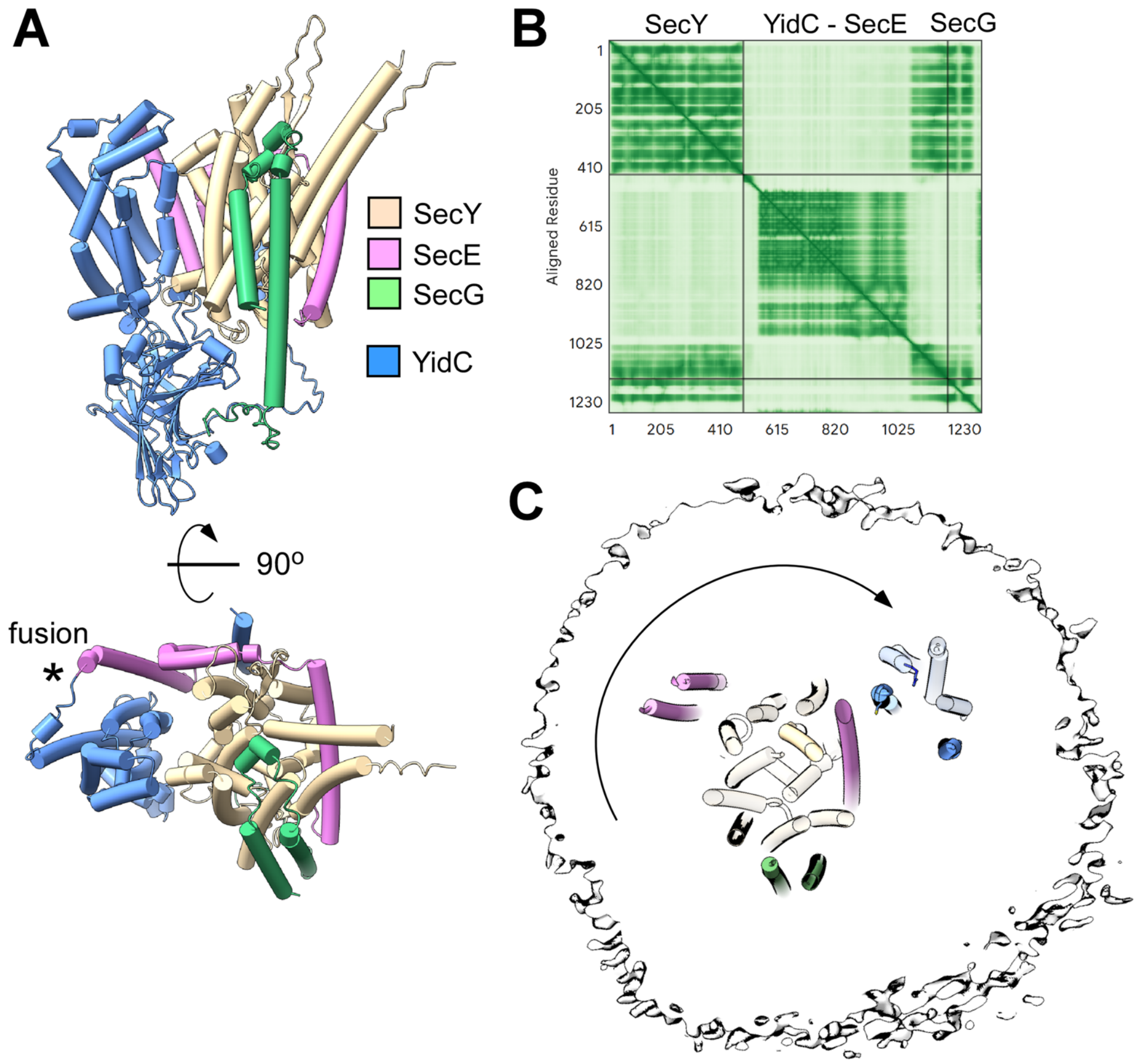

Figure EV3. Repositioning of YidC within the nanodisc upon the assembly with SecYEG.

(**A**) AlphaFold3-based model of SecYEG-YidC with the genetically fused YidC-SecE, as employed in reconstitution. YidC is positioned at the lateral gate. (**B**) PAE plot for the model shown in (**A**) suggests low confidence for SecY-YidC interactions. (**C**) Cross-section of MSP2N2 nanodisc shows the available lipid-filled space around SecYEG-YidC, allowing for YidC diffusion.

© The Author(s)

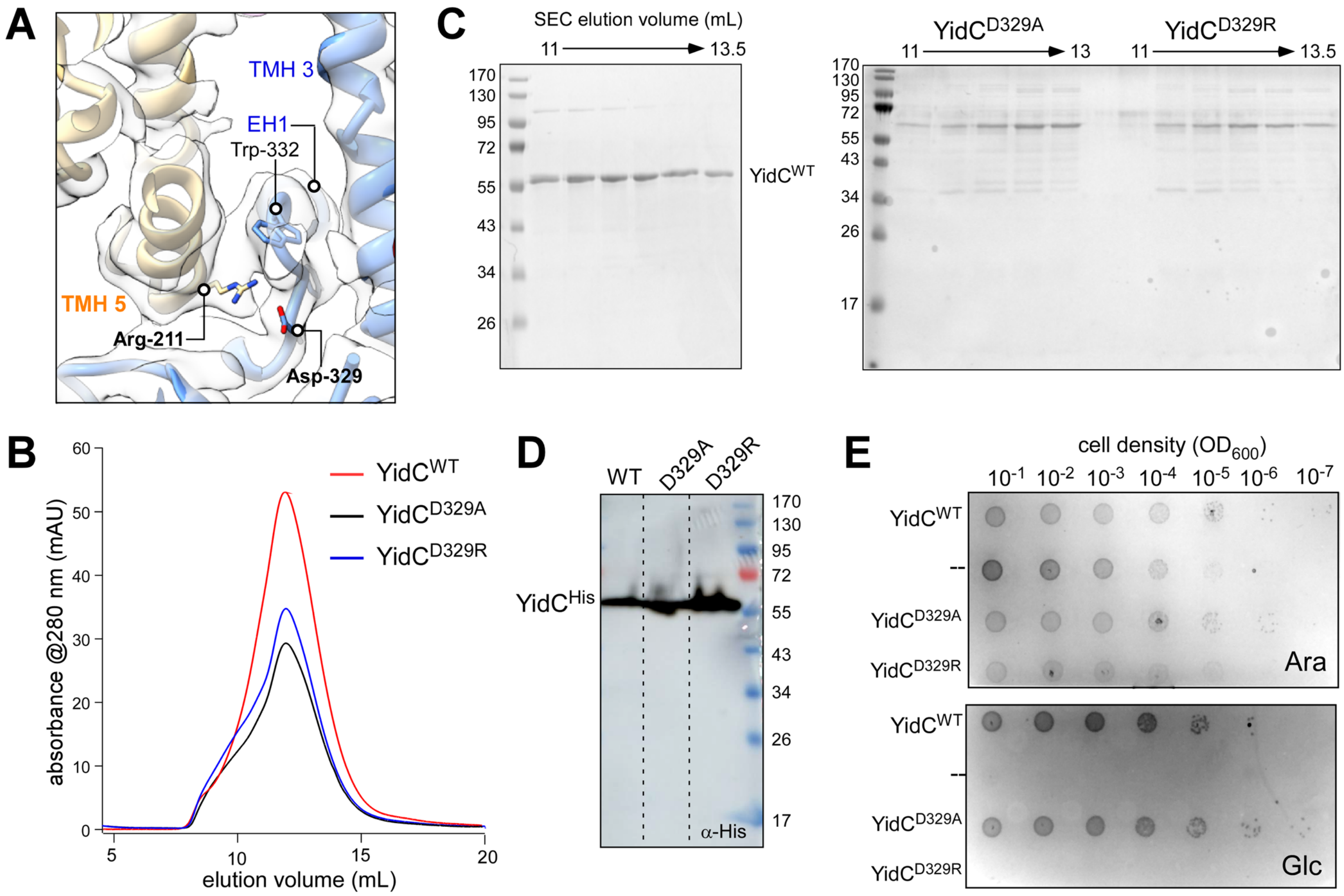

Figure EV4. Charge inversion at YidC position 329 is deleterious for the cells.

(**A**) View focusing on the periplasmic SecY-YidC interface displaying the molecular model of the SecY-YidC assembly fitted into the cryo-EM map (transparent). Proximate residues SecY Arg-211 and YidC Asp-329 are indicated in bold. (**B**) Size exclusion chromatography profiles of the DDM-solubilized and purified YidC variants. (**C**) SDS–PAGE of the size exclusion chromatography fractions shown in (**B**). (**D**) Immunodetection of the poly-histidine-tagged YidC variants expressed in *E. coli* FTL10 under permissive conditions (0.2% arabinose, 37 °C). (**E**) Complementation assay to test the effect of mutations at position 329 of YidC under permissive (0.2% arabinose, "Ara") and the complementation (0.2% glucose, "Glc") conditions. The assay was performed at 37 °C. Source data are available online for this figure.

 © The Author(s)

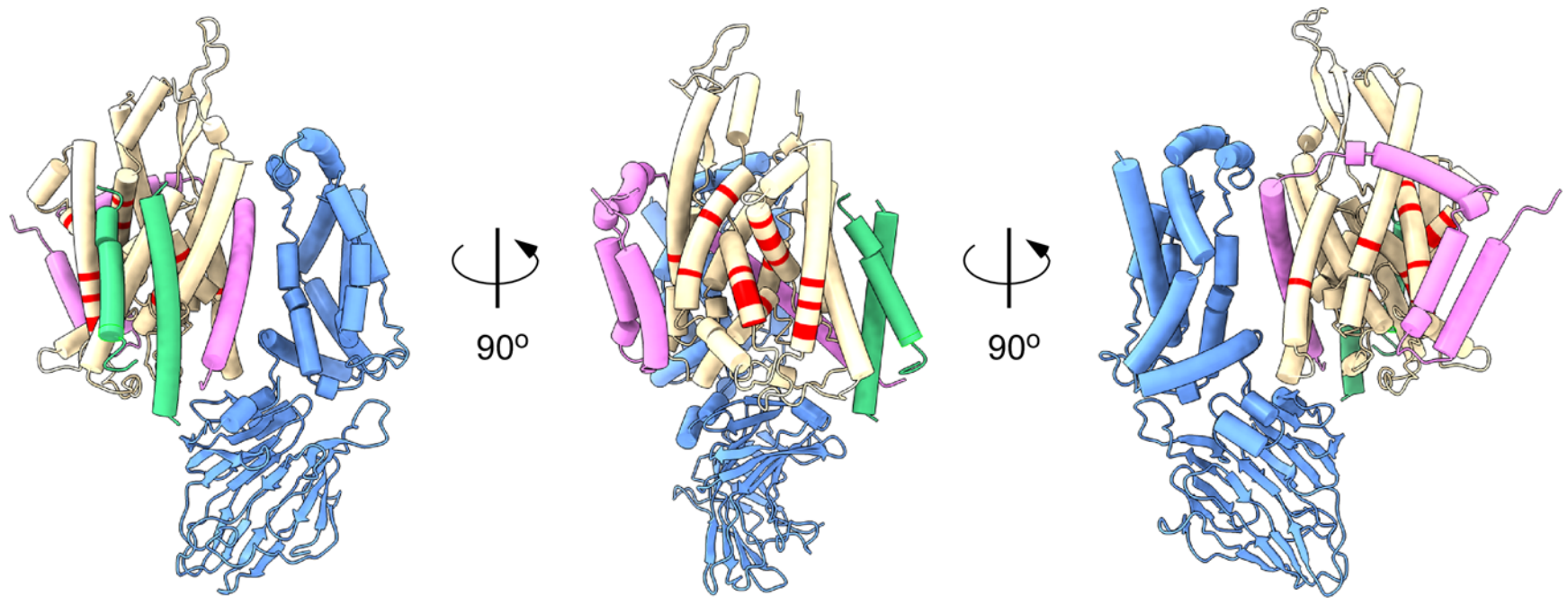

Figure EV5. Overview of the site-specific cross-linking positions to probe SecYEG-YidC interactions.

Positions employed to introduce photo-cross-linkers within SecYEG in earlier studies are shown in red (Jauss et al, 2019; Petriman et al, 2018; Sachelaru et al, 2013).
