# Supplementary material for: Substrate-induced assembly and functional mechanism of the membrane protein insertase SecYEG-YidC
Source: EMBO J. 2026 Jun 26;45(15):5457–81. doi: 10.1038/s44318-026-00837-6 (PMC13434473; doi:10.1038/s44318-026-00837-6)
Supplement: Supplementary file 3 [file 44318_2026_837_MOESM2_ESM.pdf]

THE EMBO JOURNAL

Substrate-induced assembly and functional mechanism of the membrane protein insertase SecYEG-YidC

Max Busch, Cristian Rosales-Hernandez, Michael Kamel, Yulia Schaumkessel, Eli van der Sluis, Otto Berninghausen, Thomas Becker, Roland Beckmann, and Alexej Kedrov

Corresponding author(s): Alexej Kedrov (kedrov@hhu.de) , Roland Beckmann (beckmann@genzentrum.lmu.de)

Review Timeline:

Submission Date:	24th Jan 26
Editorial Decision:	12th Mar 26
Revision Received:	9th Apr 26
Editorial Decision:	5th May 26
Revision Received:	5th May 26
Accepted:	22nd May 26

Editor: Cornelius Schneider

Transaction Report:

(Note: With the exception of the correction of typographical or spelling errors that could be a source of ambiguity, letters and reports are not edited. Depending on transfer agreements, referee reports obtained elsewhere may or may not be included in this compilation. Referee reports are anonymous unless the Referee chooses to sign their reports.)

Dear Prof. Kedrov,

Thank you for submitting your manuscript for consideration by the EMBO Journal. It has now been seen by two referees whose comments are enclosed. As you will see, both referees express interest in your manuscript and are broadly in favor of publication, pending satisfactory minor revision.

Given the referees' positive recommendations, I would like to invite you to submit a revised version of the manuscript, addressing the comments of all three reviewers. I should add that it is EMBO Journal policy to allow only a single round of revision, and acceptance of your manuscript will therefore depend on the completeness of your responses in this revised version.

When preparing your letter of response to the referees' comments, please bear in mind that this will form part of the Review Process File, and will therefore be available online to the community. For more details on our Transparent Editorial Process, please review our Editorial Policies page: https://link.springer.com/partners/embo-press/editorial-policies

We generally allow three months as standard revision time. As a matter of policy, competing manuscripts published during this period will not negatively impact on our assessment of the conceptual advance presented by your study. However, we request that you contact the editor as soon as possible upon publication of any related work, to discuss how to proceed.

Thank you for the opportunity to consider your work for publication. I look forward to your revision.

Yours sincerely,

Cornelius Schneider, PhD
Editor
The EMBO Journal
c.schneider@embojournal.org

Instructions for preparing your revised manuscript:

Read our guidance for manuscript revisions and related editorial policies: https://link.springer.com/journal/44318/submission-guidelines#cms-Revised-submissions

Please also check that the title and abstract of the manuscript are brief, yet explicit, even to non-specialists.

When assembling figures, please refer to our figure preparation guideline in order to ensure proper formatting and readability in print as well as on screen:
https://media.springernature.com/original/springer-cms/rest/v1/content/27825798/data/v1

IMPORTANT: When you send the revision we will require
- a point-by-point response to the referees' comments, with a detailed description of the changes made (as a word file).
- a word file of the manuscript text.
- individual production quality figure files (one file per figure)
- a complete author checklist
- Expanded View files (replacing Supplementary Information)
- a Reagents and Tools Table as part of the Methods section

Please remember: Digital image enhancement is acceptable practice, as long as it accurately represents the original data and conforms to community standards. If a figure has been subjected to significant electronic manipulation, this must be noted in the figure legend or in the 'Methods' section. The editors reserve the right to request original versions of figures and the original images that were used to assemble the figure.

We realize that it is difficult to revise to a specific deadline. In the interest of protecting the conceptual advance provided by the work, we recommend a revision within 3 months (10th Jun 2026). Please discuss the revision progress ahead of this time with the editor if you require more time to complete the revisions. Use the link below to submit your revision:

https://emboj.msubmit.net/cgi-bin/main.plex

Referee #1:

This manuscript provides a clear structural basis for understanding how SecYEG and YidC work together to insert multi-pass membrane proteins into the membrane, which is a fundamental and important biological process. Although membrane protein insertion has been studied for many years, structural information that captures dynamic intermediate states at near-atomic resolution has been limited. In this respect, the present study directly addresses this important question and represents a meaningful and well-designed effort.

The authors used fusion constructs of NuoK with SecM of different lengths to generate stalled, cotranslational intermediate states. These intermediates were analyzed by cryo-electron microscopy. This approach allowed the authors to capture defined stages of membrane insertion and to provide structural information about the relative positions and interactions of SecYEG and YidC. The reconstitution of these complexes and their high-resolution structural analysis are technically demanding. The overall quality of the structural data appears to be high.

Importantly, the study shows that YidC can be positioned at the backside of SecY during membrane insertion. This observation adds a new perspective to the current understanding of bacterial membrane protein insertion. Therefore, the study makes a valuable contribution to the field.

An earlier version of this work was posted on bioRxiv. The present manuscript appears to have been carefully revised since then, likely after peer review. The overall quality of the manuscript is high. The data presentation and discussion are clear and well organized. At this stage, I have no major comments.

Minor comments:
1. It would be useful to include a table summarizing the cryo-EM data collection and refinement statistics in the Supplementary Information.
2. When comparing the current structures with previously reported structures, the corresponding PDB IDs should be clearly indicated in the figure legends. Please add the missing PDB IDs where necessary.

Referee #2:

In this manuscript, the authors combined biochemistry, cryo-EM and AlphaFold predictions to characterize a bacterial co-translational SecYEG-YidC complex when engaged by the nascent membrane protein NuoK. In the bacterial translocation field, YidC has long been thought to operate at the lateral gate of SecYEG, helping to insert and chaperone transmembrane helices (TMHs) as they exit the Sec translocation channel. Recent work in mammalian and yeast systems have found that a substantial proportion of membrane insertion occurs independently of the Sec lateral gate, instead occurring "behind" Sec via other factors, some of which are evolutionarily related to YidC. The authors set out to determine whether in bacteria, a similar "back-of-Sec" mechanism of insertion might also operate. Their findings show that YidC is recruited to SecYEG in response to the substrate NuoK, and that nascent insertion intermediates of NuoK are observed behind a closed SecYEG adjacent to YidC. The structures are consistent with AlphaFold predictions of the SecYEG-YidC complexes, and reveal interesting insights into how a substrate can engage these two complexes. This suggests that the basic mechanisms of membrane insertion are broadly conserved in bacteria and eukaryotes. The work is of broad interest for EMBO Journal's readership, and will surely stimulate more work in this area. I therefore recommend publication pending some revisions to the writing.

Major points:

1) It seems that nascent NuoK is inserted in the opposite orientation in nanodiscs to what is found in native membranes. Although this is discussed at the end of the paper, with some plausible explanations provided, it is not mentioned in the results section where the strucure is described. This leads a reader to wonder what is happening and whether the findings are biologically relevant. I recommend a more direct approach to this problem by changing the following: (i) when the structure is first presented, directly and unambiguously state that the topology is backwards; (ii) mention that although this might represent an artefact with respect to NuoK, the fact that an insertion intermediate is being observed nonetheless makes the structure interesting and interpretable from the general standpoint of the mechanism of membrane insertion; (iii) tell the reader that this issue will be discussed in more detail later.

2) Similar to point 1, it is useful to be a bit clearer in the results about the fact that TMH2/3 are probably inserted shortly after termination, but that the strategy of analysing a stalled ribosome-bound intermediate helps one to visualise the likely insertion process structurally. This way, a reader is not left until the end of the paper to realise what has been done. The authors may want to mention whether they think that insertion occurs before or after the ribosome dissociates from SecYEG under normal conditions. I feel it probably occurs before the ribosome dissociates, meaning that the structures that are seen are valid proxies of the physiologic pathway, but it remains possible that insertion occurs via YidC after the ribosome and substrate have

dissociated from SecYEG. Both possibilities should probably be mentioned.

3) The interpretation that the conserved Arg is not involved in insertion is questionable. The position of TMH2 in this intermediate likely represents an endpoint location, and it is not possible to infer the path TMH2 took to reach this point. It is formally possible that Arg366 is on that path, especially considering that previous work tested its importance by growth assay (PMID: 25466392), simulation (PMID: 34873152), and other methods (such as PMID: 35148995).

4) NuoK-86 should have 36 amino acids between the site of stalling and TMH3, which is enough to span the entire ribosome exit tunnel and more. Yet, half of TMH3 is proposed to be inside the exit tunnel. I am not entirely sure why this is the case. Is it possible that the TMH assigned to TMH2 of NuoK could be TMH3 in the correct topology, and that TMH2 is what is barely visible in the density, and TMH1 is not visible? This would reconcile the opposite topology problem. In other words, how certain are the authors that their model for the substrate is correct given the rather modest resolution? Some explanation and discussion of this point might be useful.

Minor points:

Pg. 2, Line 9 - "leaves the translocon" is not quite right since it implies that the nascent chain passes from inside SecYEG through the back wall, which is of course not what seems to happen. Rephrase. Perhaps "leave the cytosolic vestibule of SecYEG" or something like that.

Pg. 2, Line 14 - The insertase machinery is not elusive; YidC and SecY have been known for decades. May want to delete "elusive"

Pg. 2, Line 14 - "prove" is too strong; perhaps "suggest an evolutionary conservation..." is better.

Pg. 3 Line 2 - IMPs do not constitute 30% of the genome content; they constitute 30% of the protein-coding genes. Genomes have lots of other content. Also, better not to equate protein with nucleic acids, so could instead say 30% of protein coding genes encode IMPs...

Pg. 14, line 32 - Sup. Fig. 15 should be 16.

Referee #1:

This manuscript provides a clear structural basis for understanding how SecYEG and YidC work together to insert multi-pass membrane proteins into the membrane, which is a fundamental and important biological process. Although membrane protein insertion has been studied for many years, structural information that captures dynamic intermediate states at near-atomic resolution has been limited. In this respect, the present study directly addresses this important question and represents a meaningful and well-designed effort.

The authors used fusion constructs of NuoK with SecM of different lengths to generate stalled, cotranslational intermediate states. These intermediates were analyzed by cryo-electron microscopy. This approach allowed the authors to capture defined stages of membrane insertion and to provide structural information about the relative positions and interactions of SecYEG and YidC. The reconstitution of these complexes and their high-resolution structural analysis are technically demanding. The overall quality of the structural data appears to be high.

Importantly, the study shows that YidC can be positioned at the backside of SecY during membrane insertion. This observation adds a new perspective to the current understanding of bacterial membrane protein insertion. Therefore, the study makes a valuable contribution to the field.

An earlier version of this work was posted on bioRxiv. The present manuscript appears to have been carefully revised since then, likely after peer review. The overall quality of the manuscript is high. The data presentation and discussion are clear and well organized. At this stage, I have no major comments.

Authors: We thank the Referee for the interest in our work and for the positive evaluation of the manuscript in its current form!

Minor comments:

Referee 1, Comment 1: It would be useful to include a table summarizing the cryo-EM data collection and refinement statistics in the Supplementary Information.

Authors: We apologize for leaving out the table from the original submission! The information is included in the revised version (Appendix Table S2).

Referee 1, Comment 2: When comparing the current structures with previously reported structures, the corresponding PDB IDs should be clearly indicated in the figure legends. Please add the missing PDB IDs where necessary.

Authors: Thank you for the point! The missing ID’s have been added to the legends of the Figure 3E and Figure EV1B (former Suppl. Figure 2B).

Referee #2:

In this manuscript, the authors combined biochemistry, cryo-EM and AlphaFold predictions to characterize a bacterial co-translational SecYEG-YidC complex when engaged by the nascent membrane protein NuoK. In the bacterial translocation field, YidC has long been thought to operate at the lateral gate of SecYEG, helping to insert and chaperone transmembrane helices (TMHs) as they exit the Sec translocation channel. Recent work in mammalian and yeast systems have found that a substantial proportion of membrane insertion occurs independently of the Sec lateral gate, instead occurring "behind" Sec via other factors, some of which are evolutionarily related to YidC. The authors set out to determine whether in bacteria, a similar "back-of-Sec" mechanism of insertion might also operate. Their findings show that YidC is recruited to SecYEG in response to the substrate NuoK, and that nascent insertion intermediates of NuoK are observed behind a closed SecYEG adjacent to YidC. The structures are consistent with AlphaFold predictions of the SecYEG-YidC complexes, and reveal interesting insights into how a substrate can engage these two complexes. This suggests that the basic mechanisms of membrane insertion are broadly conserved in bacteria and eukaryotes. The work is of broad interest for EMBO Journal's readership, and will surely stimulate more work in this area. I therefore recommend publication pending some revisions to the writing.

Authors: We thank the Referee for recognizing the impact of our study, and the constructive suggestions for the revision!

Major points:

Referee 2, Comment 1: It seems that nascent NuoK is inserted in the opposite orientation in nanodiscs to what is found in native membranes. Although this is discussed at the end of the paper, with some plausible explanations provided, it is not mentioned in the results section where the structure is described. This leads a reader to wonder what is happening and whether the findings are biologically relevant. I recommend a more direct approach to this problem by changing the following: (i) when the structure is first presented, directly and unambiguously state that the topology is backwards; (ii) mention that although this might represent an artefact with respect to NuoK, the fact that an insertion intermediate is being observed nonetheless makes the structure interesting and interpretable from the general standpoint of the mechanism of membrane insertion; (iii) tell the reader that this issue will be discussed in more detail later.

Authors: Thank you for the suggestion. We totally agree with the Referee that despite the unexpected orientation of the specific nascent protein, the identified insertion route via SecYEG-YidC may be taken by many other clients, in agreement with the recent work of Keenan and co-workers (PMID 41116047), so the visualization of the nascent chain path represents the major impact of the study. For the revised manuscript, we have followed the suggestion of the Reviewer and indicated the inverted topology of NuoK in the early part of the Results chapter (page 7), where we also suggest the general importance of the insertion route available for various clients.

Referee 2, Comment 2a: Similar to point 1, it is useful to be a bit clearer in the results about the fact that TMH2/3 are probably inserted shortly after termination, but that the strategy of analysing a stalled ribosome-bound intermediate helps one to visualise the likely insertion

process structurally. This way, a reader is not left until the end of the paper to realise what has been done.

Authors: Thank you for considering this aspect. Recognizing the differences between the naturally occurring insertion and the stalled intermediate employed for the structural analysis is indeed highly important, so we explain those early in the Results section, when introducing the in vitro system (page 6). We revised the text (lines 19-23) to make this point clear.

Referee 2, Comment 2b: The authors may want to mention whether they think that insertion occurs before or after the ribosome dissociates from SecYEG under normal conditions. I feel it probably occurs before the ribosome dissociates, meaning that the structures that are seen are valid proxies of the physiologic pathway, but it remains possible that insertion occurs via YidC after the ribosome and substrate have dissociated from SecYEG. Both possibilities should probably be mentioned.

Authors: We thank the Referee for the suggestion. Insertion of the NuoK TMH 2 should occur in a co-translational manner, and the event is illustrated by the NuoK-70 construct in our work (pages 10-11, Figure 6A-C). In respect to NuoK TMH 3, we would like to avoid the speculation/over-interpretation of the structural data. In the Discussion chapter (page 15, lines 23-32), we refer to the recent studies which show that YidC-type insertases are involved in post-translational insertion of C-terminal helices, which is indeed likely scenario for NuoK (also included in Figure 8). It is indeed possible that the ribosome remains bound to SecYEG at that stage, but it is unlikely to be involved in routing of the released polypeptide chain.

Referee 2, Comment 3: The interpretation that the conserved Arg is not involved in insertion is questionable. The position of TMH2 in this intermediate likely represents an endpoint location, and it is not possible to infer the path TMH2 took to reach this point. It is formally possible that Arg366 is on that path, especially considering that previous work tested its importance by growth assay (PMID: 25466392), simulation (PMID: 34873152), and other methods (such as PMID: 35148995).

Authors: We agree with the Referee and modify the text accordingly to indicate possible interactions of Arg-366 with NuoK at different insertion stage (page 10, lines 19-21). Furthermore, the legend of Figure 5C has been re-phrased to avoid mis-/over-interpretation.

Referee 2, Comment 4: NuoK-86 should have 36 amino acids between the site of stalling and TMH3, which is enough to span the entire ribosome exit tunnel and more. Yet, half of TMH3 is proposed to be inside the exit tunnel. I am not entirely sure why this is the case. Is it possible that the TMH assigned to TMH2 of NuoK could be TMH3 in the correct topology, and that TMH2 is what is barely visible in the density, and TMH1 is not visible? This would reconcile the opposite topology problem. In other words, how certain are the authors that their model for the substrate is correct given the rather modest resolution? Some explanation and discussion of this point might be useful.

Authors: The Referee is absolutely correct in respect to the length of the nascent chain residing within the ribosomal exit tunnel: The helices of NuoK are fully exposed from the ribosome, as explained in the experiment design (page 5, lines 13-16) and confirmed by the cryo-EM data (Figure 4A). The resolution of the nascent chain within the tunnel is below 3 A and it allows visualization and counting of individual residues to the mouth of the tunnel, where the flexible glycine-rich linker is positioned (summarized on page 9, lines 8-19; Figure 4 A and B). The chain formed a continuous density with the folded alpha-helix starting from the tunnel mouth. As we could not identify additional densities under the tunnel, which could indicate

unaccounted parts of the nascent chain, the helix was assigned to NuoK TMH 3 with high confidence.

Minor points:

Pg. 2, Line 9 - "leaves the translocon" is not quite right since it implies that the nascent chain passes from inside SecYEG through the back wall, which is of course not what seems to happen. Rephrase. Perhaps "leave the cytosolic vestibule of SecYEG" or something like that.

Authors: Thank you for the correction. We have modified the sentence, hoping to resolve this ambiguity, while remaining within the size limit of the abstract, i.e. 175 words,

Pg. 2, Line 14 - The insertase machinery is not elusive; YidC and SecY have been known for decades. May want to delete "elusive"

Authors: Done!

Pg. 2, Line 14 - "prove" is too strong; perhaps "suggest an evolutionary conservation..." is better.

Authors: Done!

Pg. 3 Line 2 - IMPs do not constitute 30% of the genome content; they constitute 30% of the protein-coding genes. Genomes have lots of other content. Also, better not to equate protein with nucleic acids, so could instead say 30% of protein coding genes encode IMPs...

Authors: The text has been modified accordingly.

Pg. 14, line 32 - Sup. Fig. 15 should be 16.

Authors: Thank you for the correction! In the revised version, the figure appears as Figure EV5.

1st Revision - Editorial Decision **5th May 2026**

Dear Prof. Kedrov,

Thank you for submitting a revised version of your manuscript which we find has addressed the concerns raised by both referees. There remain only a few mainly editorial points that have to be addressed before I can extend formal acceptance of the manuscript:

- As we are switching from a free-text author contribution statement towards a more formal statement based on Contributor Role Taxonomy (CRediT) terms, please remove the present Author Contribution section and instead specify each author's contribution(s) directly in the Author Information page of our submission system during upload of the final manuscript. See https://casrai.org/credit/ for more information.

- FIGURE CALLOUTS: all callouts should be listed sequentially; missing callouts for Fig. 7A, 7C and Dataset EV2

- APPENDIX 1 FILE WITH ToC: Appendix file needs to be in PDF format; title page should contain "Appendix for + ms title" and ToC with the page numbers for the listed items; nomenclature correct; Appendix tables should be included in Appendix PDF with the nomenclature Appendix Table S1-S2 and listed in ToC

- Please provide suggestions for a short 'blurb' text prefacing and summing up the conceptual aspect of the study in two sentences (max. 250 characters), followed by 3-5 one-sentence 'bullet points' with brief factual statements of key results of the paper; they will form the basis of an editor-written 'Synopsis' accompanying the online version of the article. Please also provide an altered synopsis image, making sure that the aspect ratio conforms to our website's format - it should be exactly 550 pixels wide and 300 pixels high.

- Figure reuse between Figure 5B and Appendix Figure S8B.
Not stated in the figure legend. If purposeful re-use of the image has occurred, please state this clearly in the figure legend.

- Sections need to be named and the order should be corrected: Title page - Abstract - Introduction - Results - Discussion - Methods - Data Availability - Acknowledgements - Disclosure and Competing Interests Statement - References - Figure Legends - Table(s) - Expanded View Figure Legends.

Please let me know if you have any questions regarding any of these points. You can use the link below to upload the revised files.
Thank you again for giving us the chance to consider your manuscript for The EMBO Journal. I look forward to receiving the final version.

With best regards,

Cornelius Schneider

____

Cornelius Schneider, PhD
Editor | The EMBO Journal
c.schneider@embojournal.org

Please refer to our figure preparation guideline in order to ensure proper formatting and readability in print as well as on screen:

https://link.springer.com/journal/44318/submission-guidelines#cms-Figure-and-data-presentation

Use the link below to submit your revision:

https://emboj.msubmit.net/cgi-bin/main.plex

All editorial and formatting issues were resolved by the authors.

2nd Revision - Editorial Decision **22nd May 2026**

Dear Prof. Kedrov,

I am pleased to inform you that your manuscript has been accepted for publication in the EMBO Journal.

Your manuscript will be processed for publication by EMBO Press. It will be copy edited and you will receive page proofs prior to publication. Please note that you will be contacted by Springer Nature Author Services to complete licensing and payment information.

You may qualify for financial assistance for your publication charges - either via a Springer Nature fully open access agreement or an EMBO initiative. Check your eligibility: https://link.springer.com/journal/44318/how-to-publish-with-us

Should you be planning a Press Release on your article, please notify the production team via this form in order to coordinate publication and release dates: https://forms.cloud.microsoft/e/eH4A8JVP6g

If you have any questions, please do not hesitate to contact the Editorial Office. Thank you for your contribution to The EMBO Journal.

Yours sincerely,

Cornelius Schneider, PhD
Editor
The EMBO Journal
c.schneider@embojournal.org

*****
Please note that it is The EMBO Journal policy for the transcript of the editorial process (containing referee reports and your response letters) to be published as an online supplement to each paper. If you should prefer removal of any referee-only figures included in the point-by-point response(s), e.g. because they may still be used for future publication or because they have been reproduced from published work by others, please do let us know immediately via response email.
More information is available here: https://link.springer.com/partners/embo-press/editorial-policies#Peer%20review

** Click here to be directed to your login page: https://emboj.msubmit.net

EMBO Press Author Checklist

Corresponding Author Name: Alexej Kedrov	
Journal Submitted to: The EMBO Journal	
Manuscript Number: EMBOJ-2026-123670	

USEFUL LINKS FOR COMPLETING THIS FORM
The EMBO Journal - Author Guidelines
EMBO Reports - Author Guidelines
Molecular Systems Biology - Author Guidelines
EMBO Molecular Medicine - Author Guidelines

Reporting Checklist for Life Science Articles (updated January
This checklist is adapted from Materials Design Analysis Reporting (MDAR) Checklist for Authors. MDAR establishes a minimum set of requirements in transparent reporting in the life sciences (see Statement of Task: 10.31222/osf.io/9sm4x). Please follow the journal's guidelines in preparing your
Please note that a copy of this checklist will be published alongside your article.

Abridged guidelines for figures
1. Data
The data shown in figures should satisfy the following conditions:
- the data were obtained and processed according to the field's best practice and are presented to reflect the results of the experiments in an accurate and unbiased manner.
- ideally, figure panels should include only measurements that are directly comparable to each other and obtained with the same assay.
- plots include clearly labeled error bars for independent experiments and sample sizes. Unless justified, error bars should not be shown for technical
- if n<5, the individual data points from each experiment should be plotted. Any statistical test employed should be justified.
- Source Data should be included to report the data underlying figures according to the guidelines set out in the authorship guidelines on Data

2. Captions
Each figure caption should contain the following information, for each panel where they are relevant:
- a specification of the experimental system investigated (eg cell line, species name).
- the assay(s) and method(s) used to carry out the reported observations and measurements.
- an explicit mention of the biological and chemical entity(ies) that are being measured.
- an explicit mention of the biological and chemical entity(ies) that are altered/varied/perturbed in a controlled manner.
- the exact sample size (n) for each experimental group/condition, given as a number, not a range;
- a description of the sample collection allowing the reader to understand whether the samples represent technical or biological replicates (including how many animals, litters, cultures, etc.).
- a statement of how many times the experiment shown was independently replicated in the laboratory.
- definitions of statistical methods and measures:
  - common tests, such as t-test (please specify whether paired vs. unpaired), simple χ2 tests, Wilcoxon and Mann-Whitney tests, can be unambiguously identified by name only, but more complex techniques should be described in the methods section;
  - are tests one-sided or two-sided?
  - are there adjustments for multiple comparisons?
  - exact statistical test results, e.g., P values = x but not P values < x;
  - definition of 'center values' as median or average;
  - definition of error bars as s.d. or s.e.m.

Please complete ALL of the questions below.
Select "Not Applicable" only when the requested information is not relevant for your study.

Materials

Newly Created Materials	Information included in the manuscript?	In which section is the information available? (Reagents and Tools Table, Materials and Methods, Figures, Data Availability Section)
New materials and reagents need to be available; do any restrictions apply?	Yes	Reagents and tools table

Antibodies	Information included in the manuscript?	In which section is the information available? (Reagents and Tools Table, Materials and Methods, Figures, Data Availability Section)
For **antibodies** provide the following information:<br>- Commercial antibodies: RRID (if possible) or supplier name, catalogue number and or/clone number<br>- Non-commercial: RRID or citation	Yes	Reagents and tools table and in Methods section

DNA and RNA sequences	Information included in the manuscript?	In which section is the information available? (Reagents and Tools Table, Materials and Methods, Figures, Data Availability Section)
**Short novel DNA or RNA including primers, probes**: provide the sequences.	Not Applicable	

Cell materials	Information included in the manuscript?	In which section is the information available? (Reagents and Tools Table, Materials and Methods, Figures, Data Availability Section)
**Cell lines:** Provide species information, strain. Provide accession number in repository **OR** supplier name, catalog number, clone number, and/**OR** RRID.	Not Applicable	
**Primary cultures:** Provide species, strain, sex of origin, genetic modification status.	Not Applicable	
Report if the cell lines were recently **authenticated** (e.g., by STR profiling) and tested for mycoplasma contamination.	Not Applicable	

Experimental animals	Information included in the manuscript?	In which section is the information available? (Reagents and Tools Table, Materials and Methods, Figures, Data Availability Section)
**Laboratory animals or Model organisms:** Provide species, strain, sex, age, genetic modification status. Provide accession number in repository **OR** supplier name, catalog number, clone number, **OR** RRID.	Not Applicable	
**Animal observed in or captured from the field:** Provide species, sex, and age where possible.	Not Applicable	
Please detail **housing and husbandry conditions**.	Not Applicable	

Plants and microbes	Information included in the manuscript?	In which section is the information available? (Reagents and Tools Table, Materials and Methods, Figures, Data Availability Section)
**Plants:** provide species and strain, ecotype and cultivar where relevant, unique accession number if available, and source (including location for collected wild specimens).	Not Applicable	
**Microbes:** provide species and strain, unique accession number if available, and source.	Yes	Reagents and Tools table and in Methods section

Human research participants	Information included in the manuscript?	In which section is the information available? (Reagents and Tools Table, Materials and Methods, Figures, Data Availability Section)
If collected and within the bounds of privacy constraints report on age, sex and gender or ethnicity for all study participants.	Not Applicable	

Core facilities	Information included in the manuscript?	In which section is the information available? (Reagents and Tools Table, Materials and Methods, Figures, Data Availability Section)
If your work benefited from core facilities, was their service mentioned in the acknowledgments section?	Not Applicable	

Design

Study protocol	Information included in the manuscript?	In which section is the information available? (Reagents and Tools Table, Materials and Methods, Figures, Data Availability Section)
If study protocol has been **pre-registered, provide DOI in the manuscript**. For clinical trials, provide the trial registration number **OR** cite DOI.	Not Applicable	
Report the **clinical trial registration number** (at ClinicalTrials.gov or equivalent), where applicable.	Not Applicable	

Laboratory protocol	Information included in the manuscript?	In which section is the information available? (Reagents and Tools Table, Materials and Methods, Figures, Data Availability Section)
Provide DOI OR other citation details if **external detailed step-by-step protocols** are available.	Not Applicable	

Experimental study design and statistics	Information included in the manuscript?	In which section is the information available? (Reagents and Tools Table, Materials and Methods, Figures, Data Availability Section)
Include a statement about **sample size** estimate even if no statistical methods were used.	Not Applicable	
Were any steps taken to minimize the effects of subjective bias when allocating animals/samples to treatment (e.g. **randomization procedure**)? If yes, have they been described?	Not Applicable	
Include a statement about **blinding** even if no blinding was done.	Not Applicable	
Describe **inclusion/exclusion criteria** if samples or animals were excluded from the analysis. Were the criteria pre-established?<br><br>If sample or data points were omitted from analysis, report if this was due to attrition or intentional exclusion and provide justification.	Not Applicable	
For every figure, are **statistical tests** justified as appropriate? Do the data meet the assumptions of the tests (e.g., normal distribution)? Describe any methods used to assess it. Is there an estimate of variation within each group of data? **I**s the variance similar between the groups that are being statistically compared?	Not Applicable	

Sample definition and in-laboratory replication	Information included in the manuscript?	In which section is the information available? (Reagents and Tools Table, Materials and Methods, Figures, Data Availability Section)
In the figure legends: state number of times the experiment was **replicated** in laboratory.	Not Applicable	
In the figure legends: define whether data describe **technical or biological replicates**.	Not Applicable	

Ethics

Ethics	Information included in the manuscript?	In which section is the information available? (Reagents and Tools Table, Materials and Methods, Figures, Data Availability Section)
Studies involving **human participants**: State details of **authority granting ethics approval** (IRB or equivalent committee(s), provide reference number for approval.	Not Applicable	
Studies involving **human participants**: Include a statement confirming that **informed consent** was obtained from all subjects and that the experiments conformed to the principles set out in the WMA Declaration of Helsinki and the Department of Health and Human Services Belmont Report.	Not Applicable	
Studies involving **human participants:** For publication of **patient photos**, include a statement confirming that consent to publish was obtained.	Not Applicable	
Studies involving experimental **animals**: State details of **authority granting ethics approval** (IRB or equivalent committee(s), provide reference number for approval. Include a statement of compliance with ethical regulations.	Not Applicable	
Studies involving **specimen and field samples:** State if relevant **permits** obtained, provide details of authority approving study; if none were required, explain why.	Not Applicable	

Dual Use Research of Concern (DURC)	Information included in the manuscript?	In which section is the information available? (Reagents and Tools Table, Materials and Methods, Figures, Data Availability Section)
Could your study fall under dual use research restrictions? Please check biosecurity documents and list of **select agents and toxins** (CDC): https://www.selectagents.gov/sat/list.htm	Not Applicable	
If you used a select agent, is the security level of the lab appropriate and reported in the manuscript?	Not Applicable	
If a study is subject to dual use research of concern regulations, is the name of the **authority granting approval and reference number** for the regulatory approval provided in the manuscript?	Not Applicable	

Reporting

The MDAR framework recommends adoption of discipline-specific guidelines, established and endorsed through community initiatives. Journals have their own policy about requiring specific guidelines and recommendations to complement MDAR.

Adherence to community standards	Information included in the manuscript?	In which section is the information available? (Reagents and Tools Table, Materials and Methods, Figures, Data Availability Section)
State if relevant guidelines or checklists (e.g., **ICMJE, MIBBI, ARRIVE, PRISMA**) have been followed or provided.	Not Applicable	
For **tumor marker prognostic studies,** we recommend that you follow the **REMARK** reporting guidelines (see link list at top right). See author guidelines, under 'Reporting Guidelines'. Please confirm you have followed these guidelines.	Not Applicable	
For **phase II and III randomized controlled trials**, please refer to the **CONSORT** flow diagram (see link list at top right) and submit the CONSORT checklist (see link list at top right) with your submission. See author guidelines, under 'Reporting Guidelines'. Please confirm you have submitted this list.	Not Applicable	

Data Availability

Data availability	Information included in the manuscript?	In which section is the information available? (Reagents and Tools Table, Materials and Methods, Figures, Data Availability Section)
Have **primary datasets** been deposited according to the journal's guidelines (see 'Data Deposition' section) and the respective accession numbers provided in the Data Availability Section?	Yes	The cryo-EM structural data have been deposited in the Electron Microscopy Data Base (EMDB) and the Protein Data Bank (PDB) repositories, additional data is inculded in Appendix dataset S1
Were **human clinical and genomic datasets** deposited in a public access-controlled repository in accordance to ethical obligations to the patients and to the applicable consent agreement?	Not Applicable	
Are **computational models** that are central and integral to a study available without restrictions in a machine-readable form? Were the relevant accession numbers or links provided?	Not Applicable	
If publicly available data were reused, provide the respective **data citations in the reference list**.	Not Applicable	
